# Mr. Pears, on Vaccine Inoculation

**Published:** 1802-03

**Authors:** C. Pears

**Affiliations:** Rockingham Row, Newington Butts


### *To the Editors of the Medical and Phyſical Journal.*

Gentlemen,

The *laſt* number of *The London Medical Review and Magazine*, having given a general invitation to its Correſpondents in the Magazine department, to tranſmit their future Commu-

---

nications to the *Medical and Phyfical Journal*, (as the former work was only to be continued as a Review) I take the liberty of availing myfelf of this offer to tranfmit the following particulars of *a Cafe of Cow-pock, which was falfly ftated in the Bills of Mortality for* 1800, *to have proved fatal*, but the fallacy of which ftatement I have been enabled to afcertain.

A feries of protracted enquiries having enabled me to trace this Cafe to Lambeth, Surry, I was favoured by the intelligent and refpectable Mr. Gawler, of that parifh, with a direction to the perfon who nurfed the child, (in China Row) and from whom I received the following account.

"The child was a fon of Mr. W. at Knightfbridge, and at the age of between four and five months was inoculated with Cow-pock, by Mr. C. of the above place. This failed, and the operation was repeated in fix weeks afterwards, and *with effect*. The puftule arofe, and proceeded in the ufual way, as to time, encruftation, &c. Mr. C. did not fee the child during this time, telling the nurfe, very properly, that it would not have any illnefs, or require any medicine. The puftule difappeared in the ufual time, and the brown encruftation was one day found in the night drefs (or bed gown) of the child, who continued in good health throughout the whole procefs, as ufual."

"TWO MONTHS *afterwards*, an eruption (fo common with infants) appeared on the chin of the child, which was "*fuppofed*" to be occafioned by an increafed flow of the faliva, in confequence of dentition, and afterwards on the lower extremities, as far as the tendo Achilles, where it was "*attributed*" to an excoriating effect of the urine; after this it became vifible on the upper extremities, the trunk of the body remaining free from it, but one of the eye-lids was alfo affected. Its appearance was that of a fcald, with a fcurfy furface; and it was "*thought*" to be of a fcorbutic nature. This eruption was "*fuppofed*" to affect the ftomach, and to occafion fpafms in that organ. It did, however, nearly difappear in about *two weeks*, and the child was thought fo well as to be reported fo to its parents.

"*At this time*, convulfions were excited by the procefs of dentition, and in a fit of this kind," *(perfectly unconnected either with the inoculation or the eruption)* "the child died."

"No idea whatever had been entertained of any connection between this child's death and the *Cow-pock*," (as indeed it would be difficult to fuppofe) "until the affertion was made by Mr. H. of F—— ftreet, Soho."

From this, therefore, arofe the error; for when the fapient Searchers came to enquire *the caufe of the child's death*, the nurfe nurfe very juftly and cautioufly anfwered, that two medical gentlemen had feen the child, and one of them faid, the death was occafioned by the *Teething*, and the other, that it was the confequence of the *Cow-pock* inoculation; from which latter affertion, as moft unfounded, but probably the moft congenial with their ideas, the report was made and entered, *for the information of the public*, and their inftruction in a lie!* So that the origin and propagation of this error arofe with Mr. H. THE CHILD DIED OF CONVULSIONS, IN CONSEQUENCE OF TEETHING, NEAR THREE MONTHS AFTER ITS INOCULATION. Of the neceffity and importance of paying a rigid adherence to TRUTH, and of having the reports made by perfons of real information and medical fcience, this example is an abundant proof. I, myfelf, can alfo witnefs how obftinate an error it produced in the minds of many individuals, who did afterwards, perhaps, feverely regret its propagation, but from whom it was with difficulty removed at the time.

Dr. Jenner, I believe, was aware of this circumftance, as he ftated it to me from the information of Dr. Ludlow, who, as the nurfe informed me, had been making the enquiry of her; but of the particulars, as above related, none I believe have yet tranfpired to make the FACT *public*. I am happy therefore in being thus enabled to communicate the *true state of the case*, and thereby to comply alfo with the requeft of Dr. J. Hoping therefore it will thus anfwer its intended purpofe, I remain,

*Rockingham Row, Newington Butts,* Your's, &c.

*Jan.* 27, 1802. C. PEARS.

---

### *On VACCINE INOCULATION.*

### By Dr. COGAN.

THE following extracts from a letter on Vaccine Inoculation, communicated by feveral medical gentlemen in Rotterdam to the Agent of the National Inftitution, appear to me not

---

* It is a well-known FACT that the report of the Searchers, however obvioufly abfurd and falfe, *cannot* be altered, nor dare any perfon *prefume* to do it. Neither is it long fince that thefe arbiters of difeafe, impudently reported the death of a Medical gentleman's child to have been occafioned by a malady the very reverfe of fact; which, when he explained, they infolently replied, that "though he was a Doctor, he knew nothing about it." Their report was received, and when the gentleman applied at the office of the parifh clerk, to have the error rectified, he was told, *it could not be done!* Excellent fource of information.

